# Using quantitative trait in adults with ADHD to test predictions of dual-process theory

**DOI:** 10.1038/s41598-020-76923-4

**Published:** 2020-11-18

**Authors:** Emil Persson, Markus Heilig, Gustav Tinghög, Andrea J. Capusan

**Affiliations:** 1 Department of Management and Engineering, Division of Economics, Linköping University, 581 83 Linköping, Sweden; 2 Center for Social and Affective Neuroscience, Department of Biomedical and Clinical Sciences, Linköping University, 581 83 Linköping, Sweden; 3 Department of Psychiatry in Linköping and Department of Biomedical and Clinical Sciences, Linköping University, 581 85 Linköping, Sweden; 4 The National Center for Priority Setting in Health Care, The Department of Health, Medicine and Caring Sciences (HMV), Linköping University, 581 83 Linköping, Sweden

**Keywords:** Human behaviour, ADHD

## Abstract

Dual-process theory is a widely utilized modelling tool in the behavioral sciences. It conceptualizes decision-making as an interaction between two types of cognitive processes, some of them fast and intuitive, others slow and reflective. We make a novel contribution to this literature by exploring differences between adults with clinically diagnosed ADHD and healthy controls for a wide range of behaviors. Given the clinical picture and nature of ADHD symptoms, we had a strong a priori reason to expect differences in intuitive vs reflective processing; and thus an unusually strong case for testing the predictions of dual-process theory. We found mixed results, with overall weaker effects than expected, except for risk taking, where individuals with ADHD showed increased domain sensitivity for gains vs losses. Some of our predictions were supported by the data but other patterns are more difficult to reconcile with theory. On balance, our results provide only limited empirical support for using dual-process theory to understand basic social and economic decision-making.

## Introduction

Attention deficit hyperactivity disorder (ADHD) in adults presents clinically with functional deficits in various aspects of life such as education, work performance, relationships, parenting, social and economic status^1–3^. These deficits are due to difficulties within three symptom clusters: (a) inattention causing careless mistakes, low frustration tolerance and problems in focusing on paperwork, reading, organizing and thinking through complex tasks; (b) hyperactivity resulting in difficulties sitting still and relaxing, tendency to work long hours or more than one job and (c) impulsivity expressed in acting and talking impulsively, overspending, frequently changing jobs and relationships, engaging in kick-seeking and/or antisocial behaviors^4^. Overall, these symptom clusters represent a reduced ability to act with forethought, and control impulsive action. A prior literature has shown that ADHD is associated with deficits in neurocognitive domains linked to executive functions such as response inhibition and working memory^4–10^. The very nature of these symptoms and deficits therefore renders individuals with ADHD ideal for testing the predictions of dual-process theory related to impulse control and emotional arousal.

Dual-process theory conceptualizes decision-making as resulting from the interaction between intuitive and reflective cognitive processes^11–17^. Intuitive processes are typically characterized as being fast, automatic, effortless, and emotional. Reflective processes, conversely, are slower and more controlled, effortful, and deliberative. The dual-process typology has shaped empirical and theoretical work in many areas of psychology and neuroscience^13,18^ and it constitutes a theoretical foundation for highly influential ideas in behavioral economics, such as the planner-doer model of Thaler and Shefrin^19^ and subsequent theoretical work on hyperbolic discounting and impulse-control problems^20,21^. It can also rationalize core results in the heuristics and biases research program, including prospect theory^14,22^. However, from an empirical point of view, substantial uncertainty remains regarding the relevance of using dual-process theory to understand decision-making, because the literature to date has been unable to establish empirical regularities that are robustly linked to behavioral disparities in intuitive vs reflective decision-making.

The goal of our paper is to examine how and to what extent intuitive decision-making influences basic social and economic decision-making that is central to understanding a wide variety of everyday behavior: (i) altruistic behavior, (ii) moral judgment, (iii) risky choices, and (iv) intertemporal choices. To this end, we develop predictions using dual-process theory in each of these four domains and subsequently test them in an experiment, by comparing the choices made by medication-naïve adults with ADHD to a healthy control group. According to a dual-process framework for understanding decision-making, poor executive control should lead individuals to rely more heavily on intuition when making decisions^13,23–25^. We therefore expect adults with ADHD to rely more heavily on intuitive cognitive processing when making decisions.

The previous experimental literature has used different types of state manipulations, for example time pressure and cognitive load or other depletion tasks, to invoke decision states thought to temporarily heighten individuals’ reliance on intuitive cognitive processing when making their decisions. Common to the four domains of decision-making under consideration in this study is that prior work has found inconsistent effects, in most cases converging toward small or insignificant findings when assessed meta-analytically or in literature reviews. An exception is the domain of moral judgment where the literature points toward a more robust link between intuition and deontological judgments^26^. For reviews, see Fromell, et al.^27^, Rand, et al.^28^, Capraro^26^ on altruistic behavior and moral judgment; and Deck and Jahedi^29^ and Drichoutis and Nayga^30^ for risky or intertemporal decision-making under cognitive load. One recent and unusually large experimental study found that time pressure seemed to increase the reflection effect of prospect theory for decisions involving risks^31^, but other studies have found different results^32^. Moreover, Lindner and Rose^33^ found that time pressure increased rather than decreased patience in intertemporal choice, which goes against the canonical prediction of dual-process theory that intuition should favor immediate rewards.

One possible explanation for the disparate findings in the previous literature is that manipulations are too weak to induce behavioral effects that are robust across studies and contexts. We circumvent this issue by exploiting variation in quantitative traits linked to ADHD, where there is a strong a priori reason to expect a differential between intuitive and reflective processing, and thus a stronger case for testing predictions of dual-process theory. This is the first study of its kind to systematically explore differences between adults with clinically diagnosed ADHD and healthy individuals for a wide range of social and economic decision tasks. Our study therefore makes a novel contribution to the growing experimental literature examining the behavioral correlates of intuitive decision-making. We also contribute directly to the medical literature that seeks to characterize decision-making in persons with ADHD. Although some studies in this field have shown that adults with ADHD have problems with reward-based decision-making^34^ and display an unusually high rate of discounting^35–37^, with difficulties in several aspects of financial decision-making^38^, the majority of findings are still based on studies with children or adolescents, and few studies consistently sampled from a medication-naïve subject pool, see e.g. the updated meta-analyses by Dekkers, et al.^39^ and Marx, et al.^40^. Studying medication naïve subjects is critical for a valid test of hypotheses about decision making in ADHD, because treatment with stimulant medications is highly effective to improve cognitive function in ADHD. In addition, there is no work we are aware of that used an experimental approach to investigate altruistic behavior or moral judgment in persons with ADHD.

## Theory and hypotheses

Dual-process theory distinguishes between two types of cognitive processing involved in reasoning and decision-making, one fast and intuitive, the other slow and reflective^11–17^. Type 1 processing is thought to be autonomous and fast. It runs without effort and makes minimal demands on working memory resources. In contrast, Type 2 processing is slow and controlled, loads heavily on working memory and supports hypothetical thinking and mental simulation^13^. Table 1 summarizes the defining features and typical correlates for each type of processing.

**Table 1:** Defining features and typical correlates of Type 1 and Type 2 processes. Source: Adapted version of Table 1 in Evans and Stanovich^13^.

Type 1 process (intuitive)	Type 2 process (reflective)
**Defining features**	**Defining features**
Does not require working memory	Requires working memory
Autonomous	Cognitive decoupling, mental simulation
**Typical correlates**	**Typical correlates**
Fast	Slow
Nonconscious	Conscious
Automatic	Controlled
Associative	Rule-based
Experience-based decision-making	Consequential decision-making

In dual-process theory, choice and judgment is a product of interaction between the two types of processing. A common view is that Type 1 processing generates default responses that may or may not be overruled by subsequent Type 2 processing^13,15,41^. Impulsivity and inattention, which are core symptoms of ADHD, are two important factors heightening decision-makers’ reliance on the default responses generated by intuitive Type 1 processing^14,15,42^. We therefore developed hypotheses for decision-making in individuals with ADHD assuming increased reliance on the intuitive features of cognitive processing.

### Altruistic behavior

The recent literature on intuitive versus reflective altruism builds on prior work that examined whether intuition promotes cooperation in social dilemmas. Rand et al. developed a theory of social heuristics, where default responses generated by intuitive Type 1 processing favor typically successful behaviors^43,44^. The central argument is that people internalize behavioral strategies that are typically advantageous in everyday life, and then use them as intuitive default responses in novel settings, for example in a lab experiment. These default responses can then be overruled by subsequent Type 2 processing, if people take the time and effort to engage in the more cognitively demanding task of calculating the optimal strategy for this particular setting. In a social dilemma, there is a tension between individually optimal behavior (selfishness) and socially optimal behavior (full cooperation) in the short term, e.g. for one-shot interactions; but in the real world, where most interactions are repeated, it is often advantageous for everyone to cooperate. Based on this we would expect individuals with heightened reliance on Type 1 processing to be more likely to cooperate, even in situations where it does not maximize individual payoffs.

In contrast to cooperation, altruism is typically not advantageous in everyday life, because it involves only unilateral giving without strategic concerns^28^. This means that intuitive default responses based on a social heuristic should be more similar to the optimal strategy that people will arrive at through subsequent Type 2 processing, and we therefore expect intuitive and reflective altruism to be largely similar^26^. This is also in line with recent meta-analyses of studies using state manipulations, e.g. time pressure or cognitive load, to induce more intuitive decision-making^27,28^.

#### Hypothesis 1

Altruistic behavior is similar for individuals with and without ADHD.

### Moral judgment

A large body of literature has examined the influence of intuitive versus reflective decision-states on moral judgment in sacrificial hypothetical dilemmas, where individuals must decide whether to harm one individual to save a greater number of individuals (see e.g. the review by Capraro^26^). The general finding in this literature is that intuitive Type 1 processing seems to be associated with deontological judgments, i.e., it is morally unacceptable to harm people for the greater good. This finding is in line with the theory of emotional engagement in moral judgment developed by Greene, et al.^45^, where automatic emotional Type 1 processing drives deontological judgments, and more effortful and controlled Type 2 processing drives consequential decision-making and utilitarian judgments^45,46^. The central idea is that our intuitive reaction to a sacrificial dilemma will characteristically center on the moral prerogative that ‘it is morally wrong to hurt an innocent person, no matter the consequences,’ and thus if we are not willing or given time to further reason about the full consequences for everyone involved, this would be our judgment. However, some people will overrule this response once they have conducted a utilitarian calculation of the pros and cons of their actions, which typically requires more controlled and effortful cognitive processing. We therefore expect that individuals with heightened reliance on Type 1 processing are more likely to make deontological judgments in sacrificial dilemmas.

#### Hypothesis 2

Individuals with ADHD are more likely to make deontological judgments in sacrificial dilemmas.

### Decisions involving risks

When choices are intuitive and fast, based on Type 1 processing, attributes related to change and difference become more important than attributes related to absolute values, because they are salient and readily accessible at the point of decision-making. This ties intuitive decision-making directly to the theoretical foundations of prospect theory, which is built around the idea that gains or losses, i.e., changes relative to a reference point, are the relevant carriers of utility, and that choices are based on decision-makers’ anticipation of the affective valence tied to these changes^14,22,47^. In contrast, expected utility theory emphasizes final states of wealth, i.e., absolute values, rather than changes, as carriers of utility, and choices are determined on the back of rational computations of expected utilities. We therefore expect that individuals with heightened reliance on Type 1 processing will make risky choices that are more in line with the predictions of prospect theory, where a central feature is that people are risk averse in the gain domain but risk seeking in the loss domain; a choice pattern known as the reflection effect.

#### Hypothesis 3

In choices between safe and risky prospects, individuals with ADHD will choose the safe option more often in the gain domain (consistent with risk aversion) but less often in the loss domain (consistent with risk seeking).

### Intertemporal choice

Intertemporal choice involves making decisions between different rewards that are distributed across time. In this context, the choices people make will typically depend on their patience, which is conceptualized as their tolerance of waiting for a given reward that is delivered at a future date. A common task used in the experimental literature involves choosing between a smaller reward delivered immediately and a larger reward delivered with delay^48^. From the perspective of dual-process theory, the relative attractiveness of these two rewards will depend on the degree to which different types of cognitive processing are engaged in the evaluation procedure. Whereas the immediate reward is salient and thus easy to evaluate via intuitive Type 1 processing, the delayed reward is more abstract and thus requires greater involvement of Type 2 processing, because some degree of mental simulation of future possibilities is needed to fully assess how useful the reward would be at some distant date^13^.

A dual-process view of intertemporal choice is supported by neuroimaging studies that map differential activation in distinct neural systems to evaluation of monetary rewards delivered at different points in time^13^. In a seminal paper, McClure, et al.^49^ found increased activation in the evolutionary old limbic and paralimbic brain systems when immediate rewards were chosen but also that activation declined rapidly as both the sooner and later rewards were delayed. In contrast, evolutionary newer prefrontal areas that are typically associated with deliberation and higher cognition showed increased activation when delayed rewards were chosen. Other studies have found similar patterns of activation during intertemporal choice^50–53^. Studies also indicate altered VS (ventral striatum) activation in reward anticipation and delivery in adults with ADHD^54^. We therefore expect increased preference for immediate rewards (and increased impatience in general) in individuals with heightened reliance on Type 1 processing.

#### Hypothesis 4

Individuals with ADHD will choose the smaller-sooner reward more often in intertemporal choices.

## Results

One hundred and eighty-four participants took up the study, n = 50 in the ADHD group and n = 134 in the control group. Both groups were similar in terms of background characteristics, including sex, age, and education (Supplementary Material Table S1). The Qb-test showed a marked hyperactivity for the adults with ADHD with 66% of the sample above 95th percentile and sample median at 99th percentile (score 2.4 points), and mean at 92% (2.2 score point, standard deviation (SD) 1.1, range – 0.4, 3.7) of expected activity for age and sex adjusted population. Visual inattention, as measured with the Qb-test showed median at 89th percentile (score 1.3 points), mean at 83rd percentile (1.2 score point, SD 0.9, range – 0.9, 3.3) compared to age and sex adjusted population values, while impulsivity measured with Qb-test had median at 70th percentile (score points 0.5), and mean at 68th percentile (1.0 score point, SD 1.5, range – 1.9, 4.3). Everyone in the ADHD group answered all questions in the study, except for a few missing responses on some of the background questions at the end of the study. In the control group, 110 participants finished the full study (see Table 2 for a comparison of background characteristics). The order of questions and realized sample size at each stage of the study can be found in Supplementary material Table S2.

**Table 2:** Sample characteristics for subjects who finished the full study.

	ADHD (n = 50)	Controls (n = 110)	Test group
Age, mean (SD), range	31.3 (8.6)	34.1 (7.5)	t(158) = 2.1, *p* = 0.04
	18–46	21–44	
Female, n (%)	28 (56%)	61 (56%)	χ^2^(1) = 0.004, *p* = 0.95
**Education**			
Elementary, n (%)	6 (12%)	13 (12%)	χ^2^(2) = 0.12, *p* = 0.94
High school, n (%)	29 (62%)	71 (64%)	-
University w/o degree, n (%)	12 (26%)	26 (24%)	-
University w degree, n (%)	0	0	-
Income (scale 1–5), mean (SD)	2.45 (1.18)	2.5 (1.23)	t(148) = 0.22, *p* = 0.82

Three subjects in the ADHD group did not answer the question about education and ten did not provide information about income.

We found no significant differences between the ADHD group and the control group on the Cognitive reflection test (CRT) or the Jellybean task. The tendency was for the ADHD group to perform worse on CRT but better on the Jellybean task. On CRT, the average number of correct responses (out of three questions) was 0.76 (SD = 0.92) in the ADHD group and 0.94 (SD = 1.11) in the control group, estimated mean difference = – 0.16, SE = 0.16, t(156) = –0.99, *p* = 0.33, 95% CI, –0.49, 0.16. On the Jellybean task, 82 percent of participants in the ADHD group and 71 percent of participants in the control group provided the correct answer, χ^2^ (1) = 2.22, *p* = 0.14, n = 160. Within the ADHD group, the correlations between Qb test score and performance on the tests were weak and insignificant (CRT, Spearman’s *ρ* = 0.11, *p* = 0.46, n = 50; Jellybean task, Spearman’s *ρ* = 0.14, *p* = 0.35, n = 50).

### Altruistic behavior

**Figure 1:**
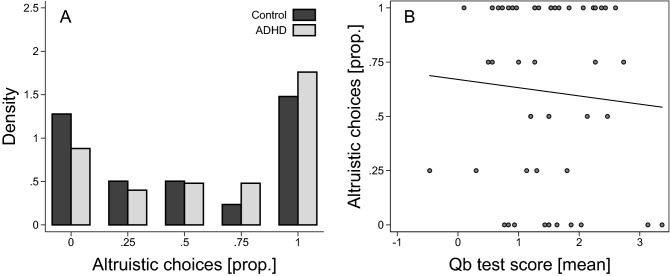
ADHD and social decision-making (binary dictator game). (**A**) Distribution of the proportion of altruistic choices (calculated for each individual) for ADHD (n = 50) and controls (n = 119). (**B**) Scatter plot of the proportion of altruistic choices and mean Qb test score for participants with ADHD (n = 50). Added line shows predicted values from a linear regression.

We found no statistically significant difference between the ADHD group and the control group on the binary dictator game (Fig. 1A). The average proportion of altruistic choices was 0.62 (SD = 0.41) in the ADHD group and 0.51 (SD = 0.43) in the control group. The estimated mean difference between individuals with and without ADHD was 11 percentage points, b = 0.11, SE = 0.07, t(165) = 1.59, *p* = 0.11, 95% CI, – 0.03, 0.25. The data is thus consistent with population effects where individuals with ADHD are at most three percentage points (lower bound on the 95% CI) more selfish than individuals without ADHD, which is a negligible difference in this context; but we cannot rule out small to moderate effects in the opposite direction. Within the ADHD group, there was no correlation between manifestation of core ADHD symptoms, measured by a standardized score on the Qb test, and altruistic behavior (Fig. 1B; Spearman’s *ρ* = – 0.05, *p* = 0.75, n = 50). Taken together, we find moderate support for the hypothesis that altruistic behavior is similar for individuals with and without ADHD.

### Moral judgment

**Figure 2:**
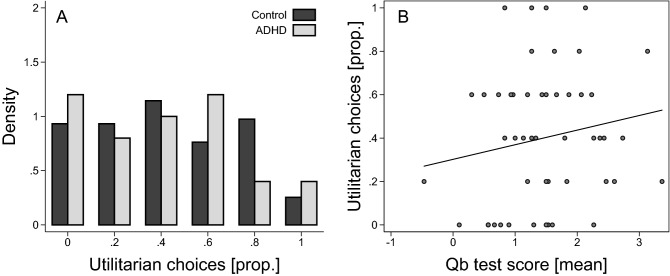
ADHD and moral judgment. (**A**) Distribution of the proportion of utilitarian choices (calculated for each individual) for ADHD (n = 50) and controls (n = 118). (**B**) Scatter plot of the proportion of utilitarian choices and mean Qb test score for participants with ADHD (n = 50). Added line shows predicted values from a linear regression.

There was no overall difference in moral judgment between the ADHD group and the control group (Fig. 2A). The average proportion of utilitarian choices was 0.4 (SD = 0.31) in the ADHD group and 0.43 (SD = 0.31) in the control group. The estimated mean difference between individuals with and without ADHD was only three percentage points, b = – 0.03, SE = 0.05, t(164) = – 0.61, *p* = 0.54, 95% CI, – 0.14, 0.07. The data are thus consistent with population effects where the difference between individuals with and without ADHD is smaller than 14 percentage points, which is a reasonably small effect in this context. Within the ADHD group, there was a small but insignificant positive correlation between Qb test score and individuals’ propensity to make utilitarian moral judgments (Fig. 2B; Spearman’s *ρ* = 0.17, *p* = 0.25, n = 50). Taken together, we find evidence against a difference in moral judgment between participants with and without ADHD, and there is thus no support for our hypothesis that individuals with ADHD are less likely to make utilitarian judgments in sacrificial dilemmas.

### Decisions involving risks

**Figure 3:**
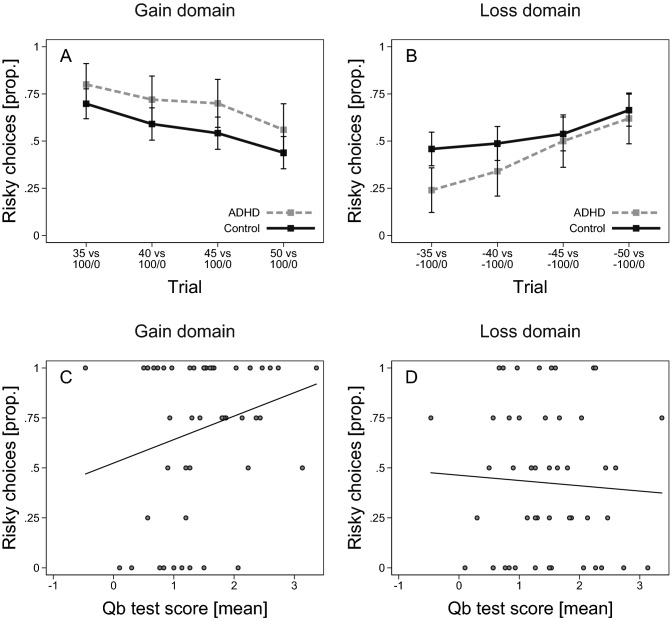
ADHD and decision-making under risk. (**A**) Proportion of participants who chose the risky alternative on a given trial (x-axis) in the gain domain, by ADHD (n = 50) and controls (n = 134). (**B**) Proportion of participants who chose the risky alternative on a given trial (x-axis) in the loss domain, by ADHD (n = 50) and controls (n = 120). (**C**) Scatter plot of the proportion of risky choices for each participant in the gain domain and mean Qb test score for participants with ADHD (n = 50). (**D**) Scatter plot of the proportion of risky choices for each participant in the loss domain and mean Qb test score for participants with ADHD (n = 50). Added line shows predicted values from a linear regression. Error bars represent 95% confidence intervals.

Participants with ADHD were more risk taking in the gain domain compared to the control group (Fig. 3A). For example, on the trial where participants chose between 35 SEK for certain and a gamble for 0 SEK or 100 SEK with equal probability, 80 percent of participants in the ADHD group and 70 percent of participants in the control group chose to gamble. The average proportion of risky choices in the gain domain was 0.70 (SD = 0.39) in the ADHD group and 0.56 (SD = 0.43) in the control group, the estimated mean difference was 13 percentage points (b = 0.13, SE = 0.07, t(180) = 1.96, *p* = 0.05, 95% CI, – 0.001, 0.27). We found a tendency for the reverse pattern in the loss domain, but the difference between the groups decreased when the magnitude of the certain loss became sufficiently large (Fig. 3B). The average proportion of risky choices was 0.43 (SD = 0.37) for ADHD participants and 0.54 (SD = 0.40) for the control group, the estimated mean difference was 13 percentage points (b = –0.13, SE = 0.06, t(166) = – 1.98, *p* = 0.05, 95% CI, – 0.25, – 0.001). Looking at the 95% CIs, we see that the data is clearly not consistent with population effects where individuals with ADHD are (i) less risk taking in the gain domain but (ii) more risk taking in the loss domain, as we had hypothesized. We reach a similar conclusion when we analyze the combined data for each individual within the ADHD group. The effects tend to go in the same direction as in the case–control comparison, but they are small and not significant. There is a weakly positive correlation between Qb test score and the propensity to take risks in the gain domain (Fig. 3C; Spearman’s *ρ* = 0.20, *p* = 0.17, n = 50), but there is no clear pattern in the loss domain (Fig. 3D; Spearman’s *ρ* = – 0.03, *p* = 0.81, n = 50).

There was, however, a comparatively strong effect within subjects, suggesting that individuals with ADHD make greater adjustments to their risk taking in the gain vs the loss domain. To summarize this effect we calculated for each individual the difference between the proportion of risky choices in the gain domain and the proportion of risky choices in the loss domain. We find that on average, for individuals with ADHD the mean (SD) difference in risk taking between gain and loss domains was 0.27 (0.44), and for participants in the control group the corresponding figure was 0.04 (0.55). Thus, there was a larger gain–loss difference within individuals in the ADHD group compared to the control group (estimated mean difference b = 0.23, SE = 0.08, t(166) = 2.70, *p* = 0.01, 95% CI, 0.06, 0.40). Similarly, within the ADHD group, there was a positive correlation between Qb test score and individuals’ propensity to take more risks in the gain domain vis-à-vis the loss domain, Spearman’s *ρ* = 0.31, *p* = 0.03, n = 50. These final results suggest increased domain sensitivity for risk taking in persons with ADHD.

### Intertemporal choice

**Figure 4:**
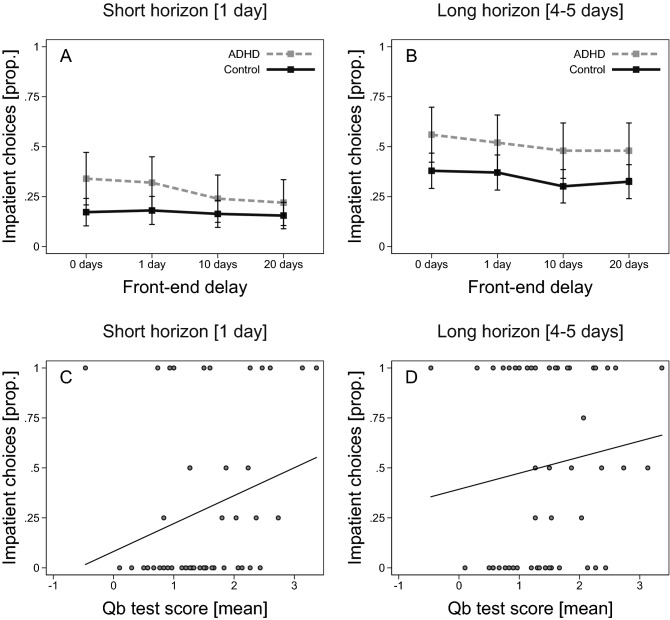
ADHD and intertemporal choices. (**A**) Proportion of participants who made an impatient choice on a given trial (x-axis) when the horizon was 1 day, by ADHD (n = 50) and controls (n = 117). (**B**) Proportion of participants who made an impatient choice on a given trial (x-axis) when the horizon was 4–5 days, by ADHD (n = 50) and controls (n = 117). (**C**) Scatter plot of the proportion of impatient choices on trials with a 1-day horizon and mean Qb test score for participants with ADHD (n = 50). (**D**) Scatter plot of the proportion of impatient choices on trials with a horizon of 4–5 days and mean Qb test score for participants with ADHD (n = 50). Added line shows predicted values from a linear regression. Error bars represent 95% confidence intervals.

Participants with ADHD were more impatient than controls (Fig. 4). The average proportion of impatient choices was 0.40 (SD = 0.38) in the ADHD group and 0.25 (SD = 0.35) in the control group, and the estimated mean difference was 13 percentage points (b = 0.13, SE = 0.06, t(163) = 2.01, *p* = 0.05, 95% CI, 0.002, 0.26). We can see in Fig. 4 that the general pattern for ADHD vs controls is similar for both horizons, 1 day and 4–5 days respectively. The figure shows the proportion of participants who made an impatient choice on a given trial. The trials are ranked (on the x-axis) according to the delay to the sooner payment, called front-end delay in the literature on intertemporal choice. For example, the second trial in Fig. 4A represents a choice between 100 SEK delivered in one day and 110 SEK delivered in two days, a trial where 32 percent of participants in the ADHD group and 18 percent of participants in the control group chose the sooner payment. Looking at the figure, panels A–B, we can also see that participants in both the ADHD group and in the control group showed (i) greater impatience in trials with a longer horizon and (ii) decreasing impatience as the delay to delivery of the sooner-smaller reward increased (see Table S5 in Supplementary material for corresponding regressions). This latter pattern is often called present bias and is consistent with hyperbolic discounting, where a characterizing feature of decision-making is that the discount rate is higher for rewards delivered closer to today.

A similar pattern of decision-making emerges when we restrict the analysis to participants in the ADHD group. There is an overall positive but insignificant correlation between Qb test scores and the proportion of impatient choices, Spearman’s *ρ* = 0.23, *p* = 0.10, n = 50. The point correlation appears to be stronger for trials with a short horizon (Fig. 4C; Spearman’s *ρ* = 0.31, *p* = 0.03, n = 50) compared to trials with a slightly longer horizon (Fig. 4D; Spearman’s *ρ* = 0.16, *p* = 0.28, n = 50), but we cannot separate these two effects statistically. Taken together, we find support for our hypothesis that individuals with ADHD would show increased impatience in intertemporal choices, but the estimated effects are weaker (noisier) than expected.

## Discussion

In this study, we used quantitative traits in adults with ADHD to test predictions of dual-process theory across several domains of social and economic decision-making. The clinical picture of adult ADHD is dominated by hyperactivity, inattention and impulse-control problems together with deficits in executive functions such as inhibition and working memory. We therefore reasoned that individuals with ADHD would rely more heavily on the intuitive features of cognitive processing when making decisions compared to a healthy control group. Overall, we find mixed results. We do confirm important aspects of our hypotheses, most notably linked to intertemporal choices and altruistic behavior. The null result for altruism was expected from a dual-process point of view and it is clearly in line with previous results in the literature that used experimental manipulations to induce intuitive versus reflective decision states^27^.

In contrast, for moral judgment and decisions involving risks we found moderate to strong evidence against our hypotheses. For moral judgment, we had expected a higher proportion of deontological judgments in individuals with ADHD, based on the theory of emotion/intuition-based moral judgment^45^ and the overall tendency in the empirical literature, reviewed in Capraro^26^. However, we observed no difference in moral judgment between individuals with ADHD and healthy controls in our data. Whereas this finding goes against some previous results in the literature, it is consistent with findings in Białek and De Neys^55^ and Tinghög, et al.^56^, who failed to confirm effects of cognitive load or time pressure on moral judgments. Our finding is also in line with the recent strand of literature on ‘utilitarian intuitions.’ Here, Bago and De Neys^57^ used a two-response paradigm and observed that most utilitarian responses materialized immediately and seemed not to require calculated deliberation, which goes against the corrective view of dual-process theory where utilitarian judgments are typically seen as products of controlled and effortful Type 2 processing.

Our results for decisions involving risks are difficult to reconcile with the findings in Kirchler, et al.^31^, who used an identical task with similar stake sizes, but instead of comparing across groups with different traits, they used time pressure to invoke intuitive decision-making. They found that time pressure led to less risk taking in the gain domain but more risk taking in the loss domain, which is consistent with the hypothesis that the reflection effect of prospect theory becomes more pronounced under intuitive decision-making. We had expected a similar effect for individuals with ADHD vis-à-vis healthy controls, but our results strongly reject such a pattern; if anything, we found the opposite tendency, where individuals with ADHD seemed to take more risks in the gain domain but fewer risks in the loss domain. A bias for risky choices in the gain domain is potentially consistent with shared dopaminergic mechanisms underlying reward valuation and impulsivity^58^, and with earlier studies indicating altered reward processing in ADHD with decreased striatal activation in reward anticipation^59,60^ and higher activation in the orbitofrontal cortex with gain outcomes^60^, suggesting that individuals with ADHD may attribute higher value to gains. These conflicting results together with the disparate findings in the previous literature using different types of manipulations, such as time pressure, cognitive load or other depletion tasks, suggest that more work is needed to better understand the interplay of cognitive processes underlying the characteristic choice patterns of prospect theory, which in itself appears to be a highly robust and replicable empirical phenomenon^61^.

No previous study used the dictator game or trolley dilemmas to characterize decision-making in adults with clinically diagnosed ADHD vis-à-vis healthy controls; we found substantial similarity in decision-making between individuals with and without ADHD on both these tasks. In contrast, risk taking in persons with ADHD has been widely studied, but this literature has largely focused on adaptive decision-making rather than decisions from description (such as standard prospect theory gambles). For example, only a handful of the studies covered by recent meta-analyses on risk taking concerned decisions from description with adult subjects, and none made a clear comparison between gain and loss domains^34,39,62^. Indeed, this is where we found the strongest link between ADHD and decision-making; individuals with ADHD made greater adjustments to their risk taking when moving from the gain domain to the loss domain, and a similar effect was found within the ADHD group when assessed based on aggregate scores on the Quantified behavioral test. Altogether, this finding suggests increased domain sensitivity for risk taking in persons with ADHD, which is a new result in the literature. Note, however, that it was based on data-contingent analyses and should not be interpreted as conclusive until replicated in future studies.

We had expected a stronger effect on the Cognitive Reflection Test (CRT), which has traditionally been used to demonstrate, and capture, differences in cognitive processing. However, an emerging literature questions whether this task is sensitive enough to reliably capture interpersonal differences in intuitive versus reflective processing-propensities^63–66^. Dual-process theories increasingly recognize that biases and fallacies in decision-making should not be attributed exclusively to intuitive processing^64,65,67^. Thus, one argument against CRT is that it conflates normative choice with intuitive thinking propensities^64,65^. Another argument against CRT comes from the observation that many people fail to solve the task even when the (allegedly intuitive) correct answer is blocked out by design, suggesting that CRT errors can be the result of many different processes and is not exclusive to a failure to engage in reflective thinking^63^. Our results are less surprising when these newer developments in the field are taken into account. They are also consistent with the finding that trait impulsivity (which is a core symptom of ADHD) is only modestly linked to performance on CRT^68^.

Our study has several strengths, including a unique sample consisting of medication naïve clinically diagnosed adults with ADHD balanced against healthy controls on age, sex and education. Adults with ADHD diagnosed in adulthood may differ from children and adolescents with ADHD^69^, which underscores the need for studies regarding decision-making in ADHD across the life-span. Participants in the control group were recruited as matched controls to around half of the participants in the ADHD group. As noted, this resulted in a balanced sample, but since not all participants with ADHD had a uniquely matched control we used a standard between-group analysis-strategy. Another strength is that we cover four different domains of decision-making and our results are therefore interesting to several distinct literatures on intuition versus reflection. The dictator game and risk tasks were incentivized but the intertemporal choice task was hypothetical. This is a limitation that might have affected our results, but it should be seen in the light of previous methodological work concluding that there is in fact little evidence of systematic differences between incentivized and hypothetical experiments on intertemporal choices, in contrast to e.g. experiments on risk taking or altruism, where incentives seem to play a larger role^48,70–72^.

Given our relatively small sample size, the results on each task should be interpreted with some caution. A priori we had 80% power to detect a medium-sized difference between the ADHD group and the control group. For the null results on altruism and morality, this implies that our results speak strongly against the existence of medium to large differences between individuals with ADHD and population controls, but it is still possible that smaller effects exist. Still, given the close link between the neurocognitive deficits commonly observed in individuals with ADHD and the core features of dual-process theory, we had a strong case for expecting relatively large behavioral differences between the groups; in particular given that all subjects in the ADHD group were medication-naïve clinically diagnosed patients. Arguably, this alleviates some of the concerns that our study was inadequately powered to detect meaningful effect sizes given the objective of our paper.

Dual-process theory is a useful and widely utilized tool for organizing our thinking about the cognitive foundations of decision-making. In its most general form, this theory (or collection of theories) characterizes decision-making as the upshot of guided interaction between different types of cognitive processes, some of them fast and intuitive, others slow and reflective. Our main idea with this paper was to broadly characterize intuitive versus reflective decision-making for a wide variety of tasks, by exploiting variation in quantitative traits linked to ADHD. Given the clinical picture and the nature of ADHD symptoms, we have a strong reason to expect heightened reliance on intuitive cognitive processing in individuals with ADHD, and we can thus infer how this influences decision-making by observing differences and similarities between individuals with and without ADHD. The fact that we found so few strong differences between these two groups supports a general conclusion that intuition versus reflection should not be seen as stable decision-making traits. Our findings are overall consistent with the trends in many of the separate literatures that used state manipulations to invoke relatively more intuitive or reflective decision-making, where effects on average seem to be small, judging by recent summaries of the literatures. Taken together, the results from our study together with these recent developments suggest that the empirical case for using a general dual-processing distinction to understand basic patterns in social and economic decision-making is weaker than previously thought.

## Materials and methods

The study was designed as a between-subject observational design comparing medication naïve adult subjects with ADHD with a healthy control group. All subjects answered anonymously on a computer. The experiment was divided into blocks associated with dependent variables. Block order was fixed but the order of the questions within each block was randomized. Before the experiment begun, subjects were informed that one of their decisions would be randomly assigned for real payment. All participants gave written informed consent. All methods were carried out in accordance with relevant guidelines and regulations. The study was approved by the Regional Ethics Review Board in Linköping, Sweden (Dnr 2014/43-31).

### Sample

Adult medication naïve patients recently diagnosed with ADHD were recruited from the outpatient department of the Psychiatric Clinic at the Linköping University Hospital. Patients were referred by their treating psychiatrists or psychologist to the research group. ADHD was screened with the Adult ADHD Self-Report Scale (ASRS), validated for screening of adult ADHD and officially translated to Swedish in 2007, with diagnosis probable at 17 points or more on either subtype scales (inattentive, hyperactive) and highly probable at 24 and above^73,74^. Clinical assessment prior to and independent of the study included interview by specialist or senior resident in psychiatry with training in diagnosing ADHD, and assessment by experienced psychologist or psychiatrist of current and childhood DSM-IV-TR (Diagnostic and Statistical Manual of Mental Disorders)^75^ symptoms and functional impairment, using DIVA (Diagnostic Interview for Adult ADHD)^76^ and structured MINI 5 interview (Mini-International Neuropsychiatric Interview)^77^. Childhood onset according to DSM-IV criteria was established based on self-report and when possible (76% of cases) also by informant report (parent, older sibling, spouse, or teacher). Diagnostic procedure also included blood sample for alcohol markers, including blood phosphatidylethanol (PEth)^78^, and drug urine screening. All patients fulfilled criteria for diagnosis and were offered ADHD medication after diagnosis and agreed to start medication after completing the study. A Qb-test (Quantified behavioral test, Copyright 2002–2011 Qbtech AB) was used as an objective measure for current symptoms of hyperactivity, impulsivity and inattention. The Qb-test combines attention measures derived from omission errors in a computer based version of the continuous performance test (CPT), impulsivity measures based on CPT commission errors, and a high resolution motion tracking system based on an infrared camera following a tracer attached to a headband the subject is wearing during assessment. Qb-test results are expressed as a score standardized around zero compared to a sex and age balanced normative Swedish population sample^79^. In our analyses we used each participant’s test score averaged over the three components, hyperactivity, inattention, and impulsivity, respectively (*mean Qb test score*).

Inclusion criteria were ADHD DSM-IV diagnosis, age 18 or older, ability to read and understand Swedish language, and signed informed consent. Exclusion criteria were concomitant autism, severe ongoing psychiatric disorder, including bipolar disorder, schizophrenia, severe obsessive–compulsive disorder (OCD); and ongoing substance-use disorder (SUD), defined as SUD within the last six months and/or positive urine drug screening or blood PEth above cut-off. Further, individuals with conditions impairing ability to understand written instruction and/or give written informed consent to the study, such as severe dyslexia, intellectual disability, as well as individuals with previous or ongoing ADHD medication (stimulants or atomoxetine), or psychoactive medication deemed to influence decision-making, such as sedatives, were excluded. Concomitant mild or moderate depression, social phobia, or generalized anxiety-disorder diagnosis with stable medication did not warrant exclusion.

A total of 59 patients, mean age 31.1 years, 44% male, were referred to the research group. Two did not meet inclusion criteria, two declined participation, two were excluded due to ongoing medication, one for ongoing substance use, and two patients did not show up for the study visit. Thus we included 50 recently diagnosed, medication naïve adults with ADHD, mean age 31.3 years, 44% male, using consecutive sampling; and 134 population controls, mean age 33.6 years, 43% male. All but three had combined ADHD subtype. The remaining three had mainly inattentive subtype, but their Qb-tests indicated significant hyperactivity/ impulsivity that did not differ from the remaining sample. Thirteen of the ADHD participants were diagnosed with comorbid anxiety disorder, four with depressive disorder (recurring, not current), two with personality disorder and two with unspecified eating disorder. Controls were recruited in collaboration with Origo Group and drawn from a sample of the general adult population previously included in their subject pool. These participants were recruited as matched controls, on age, sex, and education, to around half of the participants in the ADHD group. Our final sample yielded 80% power to detect (*p* < 0.05) a mean difference of *d* = 0.47 between the ADHD group and the control group, using a two-sided t-test, and a medium to large correlation (*ρ* = 0.39) between manifestation of core ADHD symptoms and the relevant dependent variables from our behavioral tasks.

### Behavioral tasks and procedure

The tasks included in the experiment focused on four domains of decision-making: altruistic behavior, moral judgment, risky choices, and intertemporal choices. A complete list of all tasks (including items that were included for exploratory purposes) can be found in the Supplementary materials. All participants received a show-up fee of SEK 150 (approx. 15 USD) for participation in the experiment. Participants were informed that this fee could increase or decrease depending on their decisions. At the end of the experiment, one decision was randomly selected to add or subtract from the initial sum.

Altruistic behavior was measured using a binary dictator game, where individuals chose between keeping their show-up-fee for themselves and giving it to charity. Participants were presented with four separate decisions (different charities), in randomized order. Our main dependent variable (*prop. altruistic choices*) for altruistic behavior was calculated as the proportion of choices where the participant donated their show-up fee to charity. The dictator game is a workhorse for studying fairness preferences in experiments since it involves no strategic concerns related to behavior^80,81^.

Moral judgments were measured using sacrificial moral dilemmas where subjects had to decide whether to harm a single individual in order to maximize overall good^82^. Sacrificial moral dilemmas are a classical vehicle to explore moral judgments and the conflict between utilitarian and deontological moral foundations^83,84^. In the commonly used switch dilemma, a runaway trolley is headed for five people who will be killed if it proceeds on its present course. The only way to save them is to hit a switch that will turn the trolley onto an alternate track, where it will kill one person instead of five. Pulling the switch, thereby killing the single person while saving the others, is consistent with utilitarian judgment, which implies striving toward maximization of the overall good. In contrast, not pulling the switch is consistent with deontological judgment, whereby actively causing harm to another person is morally unacceptable regardless of overall consequences. After reading each scenario, participants responded with a yes/no answer to the question “Is it morally right to [nature of action] in order to [outcome of the proposed action]”. For example, in the standard switch dilemma the question was “Is it morally right to hit the switch in order to avoid the deaths of the five men working on the tracks?” Participants were presented with five sacrificial moral dilemmas, in randomized order, and our main dependent variable (*prop. utilitarian choices*) for moral judgment was calculated as the proportion of utilitarian choices the participant made.

For decisions involving risks, we used a set of incentivized binary choices between a lottery and a safe amount of money. The lottery was identical but the safe amount varied systematically across trials^71^. Subjects made risky choices in both the gain domain and in the loss domain. In the gain domain, subjects made four choices between a 50/50-gamble (lottery) with the chance of gaining SEK 100 (appr. $10) and a certain gain that varied between SEK 35 and 50. The decisions in the loss domain were additive inverses of the decisions in the gain domain, meaning that subjects made four choices between a lottery with a 50/50-chance of losing SEK 100 and a certain loss that varied between SEK 35 and 50. The order of trials was randomized within each domain, and the gain domain was always elicited before the loss domain. Our main dependent variable (*prop. risky choices*) for each domain was calculated as the proportion of trials where the participant chose the lottery over the safe amount.

Intertemporal choice was measured using a prototypical task where subjects made repeated choices between smaller rewards delivered sooner and larger rewards delivered later^49^. In all trials, the smaller-sooner amount was fixed at SEK 100 and the larger-later amount was fixed at SEK 110. The trials differed systematically on two dimensions, (i) whether the *horizon* was 1, 4, or 5 days, and (ii) whether the smaller-sooner amount was delivered with a *front-end delay* of 0, 1, 10, or 20 days. The horizon is the delay (in days) between the sooner date of delivery and the later date of delivery, and front-end delay is the delay between today and the sooner date of delivery. For example, a trial with a 1-day horizon and a 10-day front-end delay is a choice between SEK 100 delivered in 10 days and SEK 110 delivered in 11 days. All rewards were hypothetical in this task. There were eight trials in total and they appeared in random order for each individual. Whether an individual chooses the smaller-sooner reward or the larger-later reward in a given trial depends on his or her patience, and we calculated our main dependent variable (*prop. impatient choices*) as the proportion of trials where the participant chose the smaller-sooner reward.

In addition to the four behavioral tasks explained above, we measured cognitive reflection using the Cognitive Reflection Test^85^ and the Jellybean Task^86^. The Cognitive Reflection Test involves three questions where there is an intuitive but wrong answer. For example, one of the questions was asked as follows: “A bat and a ball cost $1.10. The bat costs $1.00 more than the ball. How much does the ball cost?” Here, an intuitive answer that springs to mind quickly is that the ball costs 10 cents, but the correct answer is 5 cents. We calculate our dependent variable for this test as the number of correct answers (0–3) submitted by the participant. The Jellybean Task involves a hypothetical decision between two bowls containing 100 and 10 jellybeans respectively. Subjects are asked to imagine that they can draw one jellybean from one of the bowls, hidden behind a screen. If they draw a colored jellybean, they win five Euro. The two bowls are depicted with a label below the large bowl saying “9% colored jellybeans” and a label below the small bowl saying “10% colored jellybeans”. Here, the intuitive choice is to draw from the large bowl, as it contains a higher absolute number of colored jellybeans, but it is clearly better to draw from the small bowl because it contains a larger number of jellybeans in relative terms. Our dependent variable for the Jellybean Task is an indicator variable for choosing the smaller bowl.

### Statistical analysis

For each behavioral task in the experiment, we estimated the difference in the relevant dependent variable between the ADHD group and the control group using a linear regression with robust standard errors and controlling for age and sex. We also calculated Spearman’s rank correlation coefficient for the relevant dependent variable and participants’ average score from the Qb-test, used as a proxy measure for ADHD severity. For decisions involving risks, these analyses were conducted separately for the gain domain and the loss domain. For intertemporal choices, our main focus was on the aggregated dependent variable but we also conducted tests separately for long and short horizon. Main results from the regressions are reported in the text and more detailed information can be found in Supplementary material Table S3. All analyses were conducted using Stata version 14.2.

## Supplementary information

Supplementary Information.

## Data Availability

Analysis codes and the data used in this paper are available at the project’s OSF repository (https://osf.io/w6anv/). To ensure anonymity of participants we removed background variables (education and income) from the public data set and we transformed the age variable into a categorical variable. Results from the Qb-test were also excluded because of the sensitive nature of these data. Raw data for our main dependent variables are summarized in Supplementary Material Section S2.
