# Supplementary material for: Using quantitative trait in adults with ADHD to test predictions of dual-process theory
Source: Sci Rep. 2020 Nov 18;10:20076. doi: 10.1038/s41598-020-76923-4 (PMC7674480; doi:10.1038/s41598-020-76923-4)
Supplement: Supplementary file 1 — Supplementary Information. [file 41598_2020_76923_MOESM1_ESM.pdf]

## Supporting Information

# Using quantitative trait in adults with ADHD to test predictions of dual-process theory

Emil Persson, Markus Heilig, Gustav Tinghög, Andrea J Capusan

*S1 Additional analyzes referenced in the results section*

*S2 Summary of raw data*

*S3 Transcript of instructions for the experiment*

## *S1 Additional analyzes referenced in the results section*

**Table S1.** Sample characteristics for subjects who took up the study

| | ADHD (n=50) | Controls (n=134) | *Test group* |
|---|---|---|---|
| Age, mean (SD), range | 31.3 (8.6)<br>18–46 | 33.6 (7.7)<br>21–44 | t(182) = 1.7, *p* = .08 |
| Female, n (%) | 28 (56%) | 77 (57%) | $\chi^2(1) = .03$, *p* = .86 |
| Education | | | |
| - Elementary, n (%) | 6 (12%) | 21 (16%) | $\chi^2(2) = .35$, *p* = .84 |
| - High school, n (%) | 29 (62%) | 83 (62%) | - |
| - University w/o degree, n (%) | 12 (26%) | 30 (22%) | - |
| - University w degree, n (%) | 0 | 0 | - |
| Income (scale 1-5), mean (SD) | 2.45 (1.18) | 2.5 (1.23) | t(148) = .22, *p* = .82 |

*Note:* Three subjects in the ADHD group did not answer the question about education and ten did not provide information about income. Twenty-four subjects in the control group did not answer the question about income.

**Table S2.** Order of questions and realized sample size at each stage of the study

| | ADHD | Control |
|---|---|---|
| N took up the study | 50 | 134 |
| Risk gain, n begun (n finished) | 50 (50) | 134 (120) |
| Risk loss, n begun (n finished) | 50 (50) | 120 (119) |
| Altruism, n begun (n finished) | 50 (50) | 119 (119) |
| Moral, n begun (n finished) | 50 (50) | 118 (118) |
| Time pref. gain, n begun (n finished) | 50 (50) | 117 (116) |
| CRT, n begun (n finished) | 50 (50) | 110 (110) |
| Answered background questions on: | | |
| - age | 50 | [134] |
| - gender | 50 | [134] |
| - education | 47 | [134] |
| - income | 40 | 110 |

*Note*: We code subjects as having begun a stage if they provided at least one answer in that stage, and we use *n begun* as the final sample size for the relevant analyses. For the control group we had background information on age, gender and education beforehand, and the income question was answered at the end of the study.

**Table S3.** Main results for the behavioral tasks

| | Altruistic behavior | Moral judgment | Risk-gain | Risk-loss | Intertemporal choice |
|---|---|---|---|---|---|
| adhd | .114<br>(.071) | – .032<br>(.053) | .133<br>(.068) | –.126<br>(.063) | .127<br>(.063) |
| age | .003<br>(.004) | –.003<br>(.003) | –.002<br>(.004) | –.003<br>(.004) | –.005<br>(.004) |
| female | .164<br>(.069) | –.165<br>(.049) | –.079<br>(.070) | .080<br>(.064) | .037<br>(.056) |
| constant | .312<br>(.170) | .610<br>(.124) | .654<br>(.161) | .605<br>(.153) | .391<br>(.138) |
| observations | 169 | 168 | 184 | 170 | 167 |

*Note*: Linear regressions with robust standard errors (in parentheses). These are the underlying regressions corresponding to the results reported in the main text of the paper. Dependent variable for altruistic behavior is the proportion of choices (calculated for each individual) where the individual donated to the charity, dependent variable for moral judgment is the proportion of utilitarian choices (calculated for each individual), dependent variable for decisions involving risks, in either domain, is the proportion of choices (calculated for each individual) where the individual chose the risky alternative, and the dependent variable for intertemporal choice is the proportion of trials (calculated for each individual) where the individual chose the smaller-sooner payment.

**Table S4.** Main results for the behavioral tasks when controlling for education and income

| | Altruistic behavior | Moral judgment | Risk gain | Risk loss | Inter-temporal choice |
|---|---|---|---|---|---|
| adhd | .121<br>(.079) | – .034<br>(.059) | .161<br>(.077) | –.156<br>(.068) | .133<br>(.069) |
| age | –.001<br>(.006) | –.000<br>(.004) | .001<br>(.006) | –.006<br>(.005) | –.009<br>(.004) |
| female | .200<br>(.074) | –.150<br>(.054) | –.046<br>(.076) | .080<br>(.071) | .032<br>(.062) |
| educ., high school | –.143<br>(.131) | –.011<br>(.086) | –.120<br>(.098) | –.114<br>(.107) | .151<br>(.109) |
| educ., univ. w/o degree | .030<br>(.142) | .022<br>(.095) | –.146<br>(.117) | –.091<br>(.120) | .115<br>(.126) |
| income, 10,001–20,000 kr | –.007<br>(.102) | .073<br>(.074) | –.201<br>(.098) | .078<br>(.097) | .110<br>(.085) |
| income, 20,001–30,000 kr | .089<br>(.100) | –.029<br>(.074) | –.007<br>(.094) | .210<br>(.090) | .112<br>(.085) |
| income, 30,001–40,000 kr | .228<br>(.125) | –.099<br>(.100) | –.075<br>(.126) | .067<br>(.132) | –.005<br>(.095) |
| income, > 40,000 kr | .132<br>(.158) | .047<br>(.111) | .085<br>(.144) | .322<br>(.150) | .275<br>(.140) |
| constant | .444<br>(.208) | .528<br>(.145) | .742<br>(.191) | .702<br>(.182) | .357<br>(.150) |
| observations | 148 | 148 | 148 | 148 | 148 |

*Note*: Linear regressions with robust standard errors (in parentheses). The only difference to the results in Table S3 above is that we control for education and income and thus the sample size is lower, because some participants did not answer one or both of these questions (see Table S2). Dependent variable for altruistic behavior is the proportion of choices (calculated for each individual) where the individual donated to the charity, dependent variable for moral judgment is the proportion of utilitarian choices (calculated for each individual), dependent variable for decisions involving risks, in either domain, is the proportion of choices (calculated for each individual) where the individual chose the risky alternative, and the dependent variable for intertemporal choice is the proportion of trials (calculated for each individual) where the individual chose the smaller-sooner payment. Education and income are included as dummy variables, with “elementary school” and “0–10,000 kr (SEK)” as reference levels, respectively.

**Table S5.** Horizon effect and present bias in intertemporal choice

| | (1) | (2) | (3) |
|---|---|---|---|
| adhd | .123 (.063) | .123 (.063) | .123 (.063) |
| long horizon | | .192 (.025) | .192 (.025) |
| front-end delay | | | |
| - 1 day | | | –.009 (.016) |
| - 10 days | | | –.057 (.021) |
| - 20 days | | | –.055 (.022) |
| age | –.005 (.004) | –.005 (.004) | –.005 (.004) |
| female | .038 (.056) | .038 (.056) | .038 (.056) |
| constant | .405 (.138) | .308 (.138) | .339 (.140) |
| clusters (participants) | 167 | 167 | 167 |
| observations | 1329 | 1329 | 1329 |

*Note*: Linear regressions with standard errors (in parentheses) clustered at the level of the participant. The dependent variable is an indicator variable for choosing the smaller-sooner payment (=1 otherwise 0) in a given trial. There were eight trials (i.e., choices to be made) in this stage of the experiment.

*S2 Summary of raw data*

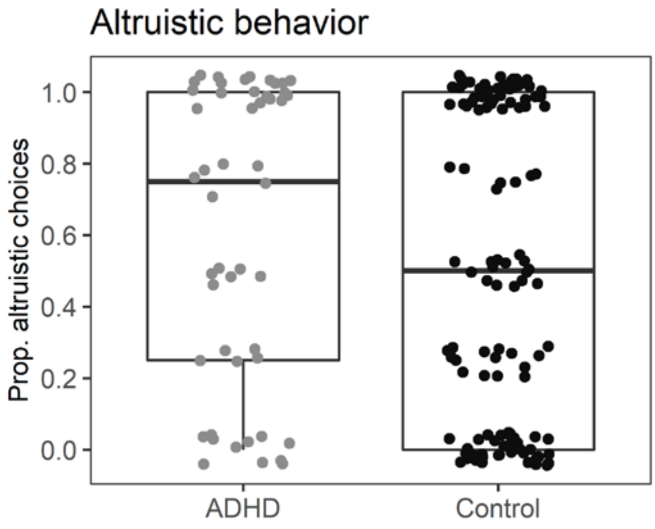


**Fig. S1.** Raw data for altruistic behavior (binary dictator game). Box- and jitter plots. The box plots show median values together with first and third quartiles; and whiskers extend to largest/smallest value (at most 1.5x IQR). One point represents one individual, n=50 for ADHD, n=119 for Control.

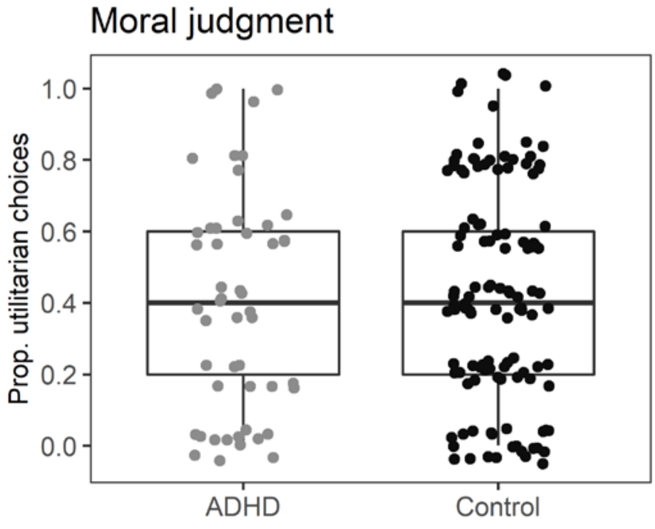


**Fig. S2.** Raw data for moral judgment. Box- and jitter plots. The box plots show median values together with first and third quartiles; and whiskers extend to largest/smallest value (at most 1.5x IQR). One point represents one individual, n=50 for ADHD, n=118 for Control.

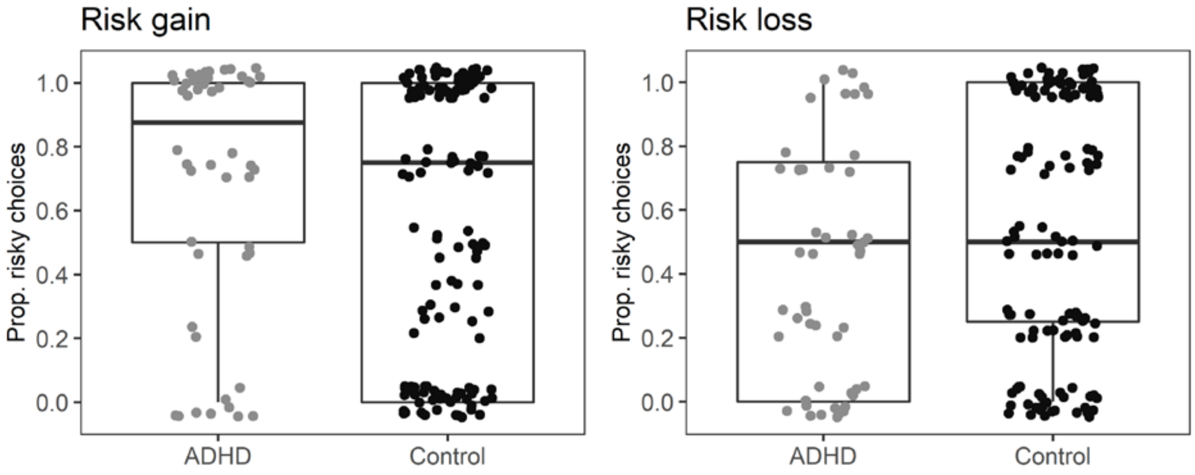


**Fig. S3.** Raw data for decisions involving risks, separated by domain. Box- and jitter plots. The box plots show median values together with first and third quartiles; and whiskers extend to largest/smallest value (at most 1.5x IQR). One point represents one individual, n=50 for ADHD and n=134 for Control in the gain domain, and n=50 for ADHD and n=120 for Control in the loss domain.

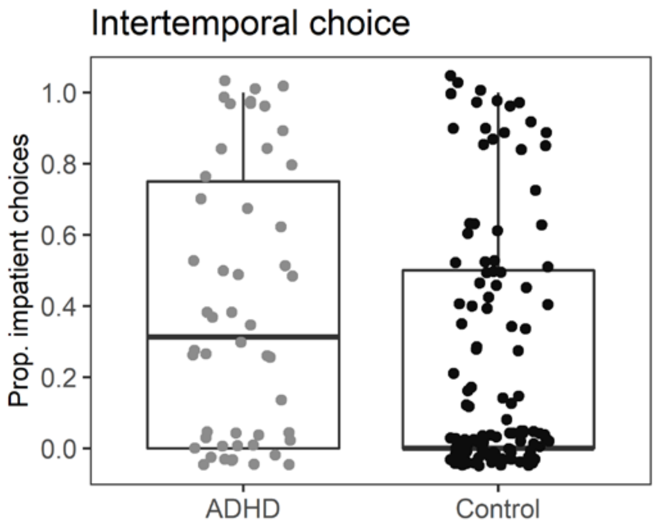


**Fig. S4.** Raw data for intertemporal choices. Box- and jitter plots. The box plots show median values together with first and third quartiles; and whiskers extend to largest/smallest value (at most 1.5x IQR). One point represents one individual, n=50 for ADHD, n=117 for Control.

**Table S6.** Distribution of CRT responses

| | ADHD | Control |
|---|---|---|
| 0/3 correct, n (%) | 25 (50%) | 56 (51%) |
| 1/3 correct, n (%) | 15 (30%) | 20 (18%) |
| 2/3 correct, n (%) | 7 (14%) | 19 (17%) |
| 3/3 correct, n (%) | 3 (6%) | 15 (14%) |

***S3 Transcript of instructions for the experiment (translated from Swedish)***

Below is a complete list of all questions asked in the experiment. Block order was fixed but the display order for items within each block was randomly chosen at the subject level.
$1 ≈ 10 Swedish kronor (SEK) at the time of writing (June 24, 2020).

**Block 1 – Risk gain**

NEW SCREEN
*In the following 4 questions, you will choose whether to receive a sum of money with certainty or to participate in a gamble. If you choose to gamble, we'll flip a coin and if the outcome is heads, you will receive an additional 100 SEK for your participation, while if the outcome is tails you will get 0 SEK additionally for your participation. This means that you have 50% chance of winning 100 SEK additionally if you choose the coin toss.*
*Press 'next' when you are ready to start.*

NEW SCREEN
*Do you choose 35 SEK with certainty or to flip a coin for 100 SEK?*

- *35 SEK with certainty*
- *Flip a coin for 100 SEK*

[There were four items in this block and the certain gain varied between 35 and 50 SEK (in 5-SEK increments).]

**Block 2 – Risk loss**

NEW SCREEN

*In the following 4 questions, you will choose whether to lose a sum of money with certainty or to participate in a gamble. If you choose to gamble, we'll flip a coin and if the outcome is heads, you will lose 0 SEK of the money you receive for your participation, while if the outcome is tails you will lose 100 SEK of the money you receive for your participation. This means that you have 50% chance of losing 100 SEK if you choose the coin toss.*

*Press 'next' when you are ready to start.*

NEW SCREEN

*Do you choose a loss of 35 SEK with certainty or to flip a coin for a loss of 100 SEK?*

- *Lose 35 SEK with certainty*
- *Flip a coin for losing 100 SEK*

[There were four items in this block and the certain loss varied between 35 and 50 SEK (in 5-SEK increments).]

**Block 3 – Altruistic behavior**

NEW SCREEN

*In the following question, you will choose how to allocate 50 SEK (in addition to the payment you receive for your participation) between yourself and an organization.*

*Press 'next' when you are ready to start.*

NEW SCREEN

*Do you choose 50 SEK for yourself or 50 SEK for BRIS?*

- *50 SEK for yourself*
- *50 SEK for BRIS*

[BRIS is a Swedish organization that supports children, Children's Rights in Society. There were four items in this block, one for each of the following charity organizations: Children's Rights in Society (BRIS), the World Wide Fund for Nature (WWF), Doctors Without Borders, and the Swedish Heart-Lung Foundation.]

**Block 4 – Moral dilemmas**

NEW SCREEN

*In the following 8 questions, different scenarios will be described. Please answer whether you think that the described action in the scenario is morally right or wrong.*

*Press 'next' when you are ready to start.*

NEW SCREEN

*Imagine that a runaway trolley is barreling down toward five workers on the railway tracks. You are standing beside the tracks, next to a switch. The only way to avoid the deaths of the five workers on the tracks is to hit the switch, thereby diverting the trolley onto a sidetrack where it will instead kill one worker.*

*Is it morally right to hit the switch to avoid the deaths of the five workers?*

- *Yes*
- *No*

NEW SCREEN

*Imagine that a runaway trolley is barreling down toward five workers on the railway tracks. You are on a footbridge over the tracks, in between the approaching trolley and the five workers. Next to you on this footbridge is a stranger who happens to be very large. The only way to avoid the deaths of the five workers is to push this stranger off the bridge and onto the tracks below where his large body will stop the trolley. The stranger will die if you do this, but the five workers will be saved.*

*Is it morally right to push the stranger onto the tracks to avoid the deaths of the five workers?*

- *Yes*
- *No*

NEW SCREEN

*Imagine that you are on a cruise ship when there is a fire on board, and the ship has to be abandoned. The lifeboats are carrying many more people than they were designed to carry. The lifeboat you're in is sitting dangerously low in the water, a few inches lower and it will sink. The seas start to get rough, and the boat begins to fill with water. If nothing is done, it will sink and everyone on board will die. However, there is an injured person who will not*

*survive in any case. If you throw that person overboard the boat will stay afloat and the remaining passengers will be saved.*
*Is it morally right to throw this person overboard to save the lives of the remaining passengers?*

- *Yes*
- *No*

NEW SCREEN
*Imagine that you are the late-night watchman in a hospital. Due to an accident in the building next door, there are deadly fumes rising up through the hospital's ventilation system. In a certain room of the hospital are three patients. In another room there is a single patient. If you do nothing the fumes will rise up into the room containing the three patients and cause their deaths. The only way to avoid the deaths of these patients is to hit a certain switch, which will cause the fumes to bypass the room containing the three patients. As a result of doing this the fumes will enter the room containing the single patient, causing his death.*
*Is it morally right to hit the switch to avoid the deaths of the three patients?*

- *Yes*
- *No*

NEW SCREEN
*Imagine that you are a civilian in a war zone where enemy soldiers have taken over your village. They have orders to kill all remaining civilians older than two years. You and some of your townspeople have sought refuge in the cellar of a large house. Your baby begins to cry loudly. You cover his mouth to block the sound. If you remove your hand from his mouth his crying will summon the attention of the soldiers who will kill you and everyone else who are hiding out in the cellar, except your child. To save yourself and the others you must smother your child to death.*
*Is it morally right to smother your child to save yourself and the other town people?*

- *Yes*
- *No*

NEW SCREEN – this item was not included for analysis (in this paper or elsewhere)

*Imagine that your close friend is moving and asks for your help. You don't feel like helping, but you know that you probably should, since your friend usually helps you out when you need it. You consider giving your friend 500 SEK instead, which would be enough to pay for someone else's help.*
*Is it morally right to pay your friend to avoid helping your friend move?*

- *Yes*
- *No*

NEW SCREEN – this item was not included for analysis (in this paper or elsewhere)
*Imagine that a smaller company is manufacturing kitchen tables. Even though the manufacturing costs have decreased 200 SEK per table, due to a decrease in the price of inputs (material), the company maintains its original retail price for the tables.*
*Is it fair to maintain the original retail price like this?*

- *Yes*
- *No*

NEW SCREEN – this item was not included for analysis (in this paper or elsewhere)
*Imagine that a hardware store is selling snow shovels for 150 SEK a piece but on the morning after a night with heavy snowfall, they raise the price to 200 SEK a piece.*
*Is it fair to raise the price like this?*

- *Yes*
- *No*

**Block 5 – Time preferences**

NEW SCREEN

*In each of the following 14 hypothetical questions, you will choose between different sums of money that are delivered at different points in time.*

*Press 'next' when you are ready to start.*

NEW SCREEN

*Do you choose 100 SEK today or 110 SEK tomorrow?*

- *100 SEK today*
- *110 SEK tomorrow*

[There were eight items of this type. They were displayed in random order and they were identical except for the dates of delivery; the sooner date was today, tomorrow, in 10 days, or in 20 days; and the horizon was either 1, 4 or 5 days. The full list of date combinations for these eight trials is: (1) today vs tomorrow, (2) today vs in 5 days, (3) tomorrow vs in 2 days, (4) tomorrow vs in 5 days, (5) in 10 days vs in 11 days, (6) in 10 days vs in 15 days, (7) in 20 days vs in 21 days, (8) in 20 days vs in 25 days.]

NEW SCREEN – this item was not included for analysis (in this paper or elsewhere)

*Suppose you could choose between two options: either you receive 1,000 SEK today or you receive a certain amount of money in one month. What amount delivered one month from today would make you indifferent between these two options?*

[There were six items of this type. None of them was included for analysis. They were displayed in random order and they were identical except for the amount of the sooner payment and the time to delivery of the later payment. The full list of date-sum combinations is: (1) 1,000 SEK today or X in 1 month, (2) 50 SEK today or X in 1 month, (3) 13,000 SEK today or X in 1 month, (4) 500 SEK today or X in 1 month, (5) 1,000 SEK today or X in 1 year, (6) 1,000 SEK today or X in 10 years.]

**Block 6 – Time preferences for saving lives**

NEW SCREEN

*In each of the following 12 hypothetical questions, you will choose between saving different numbers of persons at different points in time.*

*A new question will appear each time you press 'next'*

*Press 'next' when you are ready to start.*

NEW SCREEN – this item was not included for analysis (in this paper or elsewhere)

*Do you choose to save 100 lives today or 110 lives tomorrow?*

- *100 lives today*
- *110 lives tomorrow*

[There were eight items of this type. None of them was included for analysis. They were displayed in random order and they were identical except for the dates of saving lives; the sooner date was today, tomorrow, in 10 days, or in 20 days; and the horizon was either 1, 4 or 5 days. The full list of date combinations for these eight trials is: (1) today vs tomorrow, (2) today vs in 5 days, (3) tomorrow vs in 2 days, (4) tomorrow vs in 5 days, (5) in 10 days vs in 11 days, (6) in 10 days vs in 15 days, (7) in 20 days vs in 21 days, (8) in 20 days vs in 25 days.]

NEW SCREEN – this item was not included for analysis (in this paper or elsewhere)

*Suppose you could choose between two options: either you save 1,000 lives today or you save a certain number of lives in one month. What number of lives saved one month from today would make you indifferent between these two options?*

[There were six items of this type. None of them was included for analysis. They were displayed in random order and they were identical except for the number of lives saved at the sooner date and the time to the later date. The full list of date-number combinations is: (1) 1,000 lives today or X in 1 month, (2) 50 lives today or X in 1 month, (3) 13,000 lives today or X in 1 month, (4) 500 lives today or X in 1 month, (5) 1,000 lives today or X in 1 year, (6) 1,000 lives today or X in 10 years.]

**Block 7 – CRT & Jellybean task**

NEW SCREEN

*There are four questions in this part. Please think through your answers.*

*Press 'next' when you are ready to start.*

NEW SCREEN – CRT #1

*A bat and a ball cost 110 SEK. the bat costs 100 SEK more than the ball. How much does the ball cost?*

NEW SCREEN – CRT #2

*If it takes 5 machines 5 minutes to make 5 widgets, how long would it take 100 machines to make 100 widgets?*

NEW SCREEN – CRT #3

*In a lake, there is a patch of lily pads. Every day, the patch doubles in size. If it takes 48 days for the patch to cover the entire lake, how long would it take for the patch to cover half of the lake?*

NEW SCREEN – Jellybean task

*On the image below you can see two bowls. There are 100 jellybeans in Bowl A and 10 jellybeans in Bowl B. Your task is to pick one of the bowls. Please imagine that you will be allowed to draw (without looking) one jellybean from the bowl you picked and if you draw a red bean you will win 100 SEK!*

*Do you prefer to draw from Bowl A or B?*

- *Bowl A*
- *Bowl B*

[Image of the two bowls here]

[The three CRT items appeared in random order and the jellybean task always appeared last.]

**Block 8 – background questions**

NEW SCREEN

*Are you a man or a woman?*

- *Man*
- *Woman*

*How old are you?* [Slider]

*What is your highest educational attainment?*

- *Elementary school or similar*
- *High school*
- *University less than 3 years*
- *University 3 years or longer*

*Approximately what is your monthly income before tax? (Monthly income includes unemployment benefits and other insurance fund compensations, operating income, pensions etc., as well as other forms of benefits including for having children or studying.)*

- *0–10,000 SEK*
- *10,001–20,000 SEK*
- *20,001–30,000 SEK*
- *30,001–40,000 SEK*
- *40,001 SEK and above*
